# Epiduroscopic laser neural decompression as a treatment for migrated lumbar disc herniation: Case series

**DOI:** 10.1097/MD.0000000000010291

**Published:** 2018-04-06

**Authors:** Jinyoung Oh, Daehyun Jo

**Affiliations:** Department of Anesthesiology and Pain Medicine, Daejeon St. Mary's hospital, The Catholic University of Korea, Daejeon, Korea.

**Keywords:** Epiduroscopic laser neural decompression, epiduroscopy, indication of epiduroscopic laser neural decompression, migrated disc herniation

## Abstract

**Objective:**

Epiduroscopic laser neural decompression (ELND) is one of the more invasive techniques for managing patients with herniated lumbar disc. However, ELND can be used to treat, and diagnose the epidural pathology; indications for ELND remain controversial, especially, when applied in cases of large disc extrusion and migrated disc. This paper reports cases of patients that were satisfied with the ELND procedure for migrated lumbar disc herniation.

**Methods:**

We reviewed the medical records of patients that received ELND for migrated lumbar disc in an outpatient clinic. The patients complained of low back pain with radicular pain with an intensity over 5 on a numeric rating scale (NRS) that had persisted for over 1 month. The Magnetic resonance imaging (MRIs) showed migrated lumbar disc herniation, and patients opted for ELND because they had previously experienced nerve blocks, and did not want to receive open surgery for their pain, even after the limitations of ELND were explained.

**Results:**

Patients reported that their pain was dramatically reduced, and other discomfort symptoms, such as numbness, were also reduced after the procedure. In follow-up, all of the patients were satisfied with the results.

**Conclusion:**

We applied the ENLD procedure to mechanically, remove disc material that compressed the spinal nerve, and the patients were satisfied, and reported symptom relief. ELND was a sufficient treatment approach for lumbar migrated herniated disc for patients who did not want to undergo open spine surgery.

## 1 Introduction

Low back pain with or without leg pain is a major health issue for many patients that visit the outpatient pain clinic. Although there are many conventional options to manage this type of pain in patients, surgery was considered to be essential to treat lumbar herniated disc when conservative treatments failed. However, many minimally invasive techniques have been developed to treat patients with low back pain with or without leg pain who do not want to undergo open surgery. Epiduroscopic laser neural decompression (ELND) is a representative procedure that can treat patients with low back pain with or without leg pain under direct vision.^[1]^ ELND has several advantages: The epidural space can be viewed directly, and pathologies can be removed simultaneously in the epidural space without open surgery. Epiduroscopy has been applied for failed back surgery syndrome (FBSS), and application fields have extended as devices, and techniques have developed. Although a herniated lumbar disc is an indication of ELND, indications for ELND remain controversial, especially in cases of large disc extrusion, and migrated disc. It can be difficult, and challenging to choose the right method to treat patients with large disc extrusion, and migrated disc.^[2]^

We report very interesting cases where ELND was associated with good outcomes for patients with migrated lumbar disc who did not want to undergo open surgery, even when the limitations of ELND were explained.

## 2 Methods

This case series is based on retrospective design. Authors reviewed medical records of patients who received ELND for their low back pain with radiating leg pain from migrated disc herniation between March 2013 to 2017 in a single center which is a university hospital located in KoreaFour patients were enrolled, and their demographic data, Magnetic resonance imaging (MRI) findings of migrated disc, dose of laser used during the procedure, procedure time, pain score before and after the procedure were collected. This case series was approved by the institutional review board (IRB) of Daejeon St. Mary's Hospital (DC17RESI0071).

### 2.1 Case series

**Figure 1:**
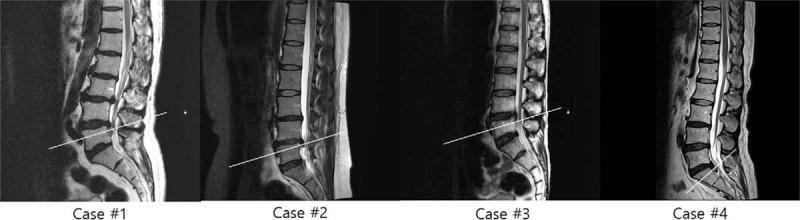
Lumbar MRIs of the patients (Sagittal view). White lines are on the level of migrated discs. MRI = Magnetic resonance imaging.

Four patients with low back pain who suffered from radiating pain rated at over 5 on the numeric rating scale (NRS) and had persisted for over 1 month visited our pain center for further interventional treatment. All patients had received conventional treatments including physical therapy, pharmacotherapy, and nerve blocks at local pain clinics. The demographic data are presented in Table 1. We confirmed the pathologic level of the associated pain by comparing history taking, and physical examination with lumbar x-ray, and MRI results. The MRIs showed lumbar disc migration (Fig. 1). We observed the size of the migrated disc fragment, which was evaluated based on MRI, and in terms of length, width, and height. The length, and width were evaluated on the axial view, on the largest cut. The height was evaluated on the sagittal view from the disc height to the lowest part of herniation (Table 2). A caudal epidural block was performed to evaluate the epidural contrast media and patient responses to nerve blocks after the laboratory blood test for blood coagulation problems. The patients were not satisfied with the caudal epidural block, and indicated that they wanted to receive ELND because they had previously received nerve blocks before visiting our clinic, and did not want to receive open surgery for their pain, even after the limitations of ELND were explained.

**Table 1:**
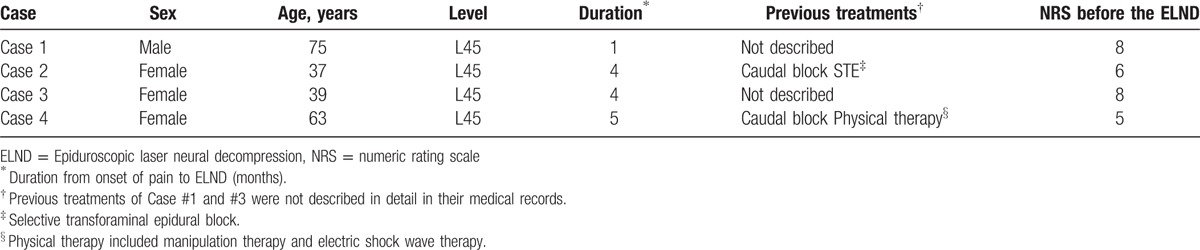
Patient demographic data.

**Table 2:**
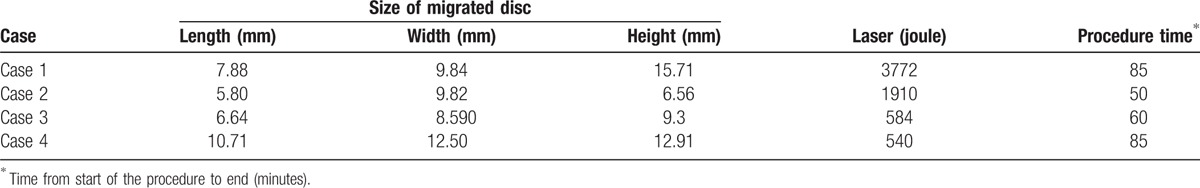
Migrated disc size, dose of laser, and procedure time.

### 2.2 Procedure

**Figure 2:**
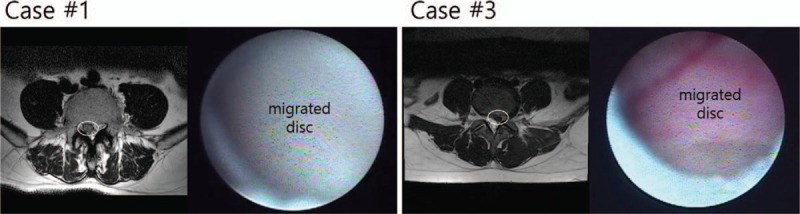
Migrated disc of Case 1 and 3 shown in MRI and epiduroscope. White circles in MRI images indicates the migrated disc. MRI = Magnetic resonance imaging.

**Figure 3:**
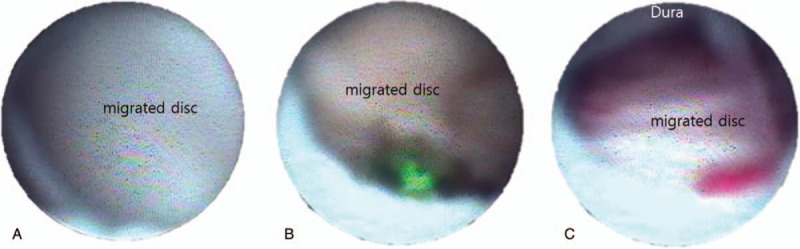
Epiduroscopic view of the anterior epidural space during the ELND. (A) Migrated disc completely obstructs the space. (B) Laser is used for firing the migrated disc. (C) After the migrated disc is collapsed, dura went down, and could be seen on the scope view. ELND = Epiduroscopic laser neural decompression.

**Figure 4:**
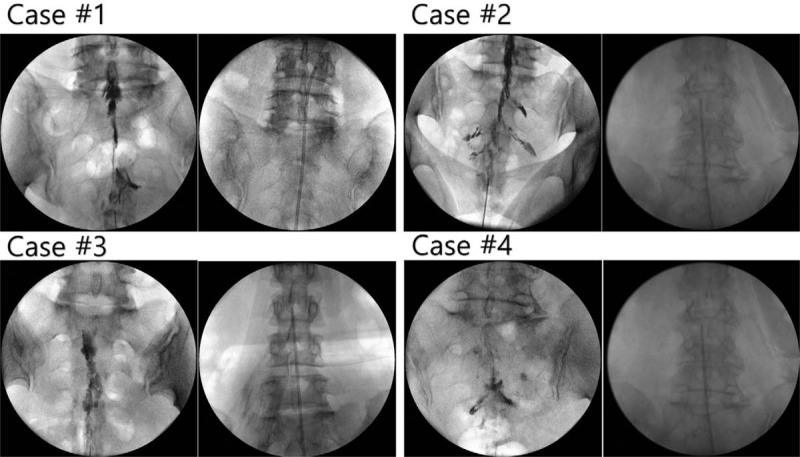
Epidurogram (left) and final catheter position during ELND (right). The catheter advanced up over the levels of migrated disc after removing the disc fragments by laser. ELND = Epiduroscopic laser neural decompression.

ELND was performed in a clean operating room after the patient provided informed consent. All procedures were performed by one expert who had experienced more than 100 cases of ELND. The patient was placed in a prone position with a pillow under the pelvis, which allowed the legs to be spread wide. After sterilization, local anesthetics were allowed to infiltrate around the sacral hiatus. Using the Seldinger technique, a cannula was inserted into the caudal epidural space, and a catheter that contained the epiduroscope (Sanat 2.8N, Intervan Inc, Seongnam-si, Korea), and laser fiber (P20, Lumenis Ltd, Yokne’am Illit, Israel) was then administered into the epidural space via the cannula. The catheter was administrated up to the level of the migrated disc in the anterior epidural space. After observing that the disc material blocked the advance of the catheter by the epiduroscope (Fig. 2), we used a Holmium:yttrium-aluminum-garnet (Ho:YAG) laser to remove the migrated disc after the lesion was confirmed by stimulation using the laser tip, and low energy laser. Lasering was performed until the upper dura by the migrated disc material in the anterior epidural space was visible with the epiduroscopic, and movable based on the saline infusion at the upper part of the scope (Fig. 3). The catheter could pass above the level of pathology (Fig. 4) after this was completed. The dose of laser, amount of saline irrigation, and operation time are described in Table 2. NRS was checked from a first visit to end of follow up by a nurse in the center.

## 3 Result

**Figure 5:**
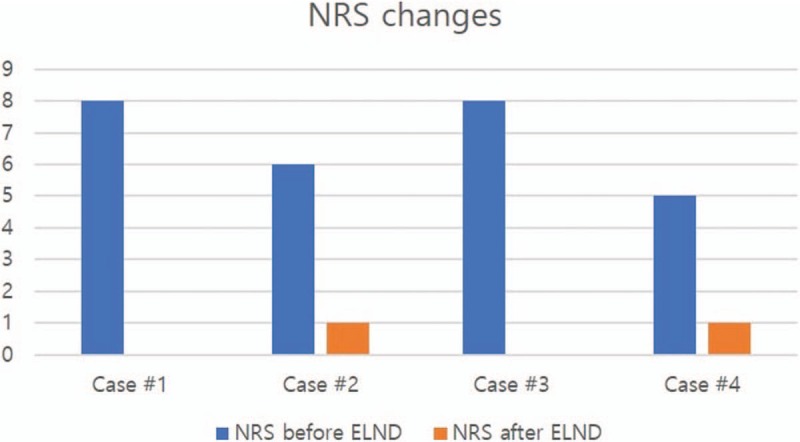
NRS changes after ELND. All patients’ pain score was dramatically reduced after the procedure. And all of them were satisfied with the results. ELND = Epiduroscopic laser neural decompression, NRS = numeric rating scale.

Patients’ migrated lumbar disc shown in MRI was confirmed by epiduroscope, and partially removed by laser during the procedure. We recorded the amount of laser that was used, in addition to the total procedure time; however, these values were not found to be correlated with the size of the migrated disc. NRS of all patients was dramatically reduced just after the procedure (Fig. 5). Furthermore, patients indicated that other discomfort symptoms, such as numbness, were also reduced. No adverse events including post-procedural infection, hematoma, and neurologic deficits had been occurred after the procedure in all cases. In case 1, the patient reported no pain for a month after the procedure, but later complained of mild pain. That pain was controllable by other interventions including intermittent epidural blocks (caudal block or selective transforminal epidural block). Other cases, post-procedure NRS was remained until the end of follow up without other interventions. The patients were taking gabapentin, and non-steroid anti-inflammatory drugs (NSAIDs) before the procedure, and maintained until the end of follow up. The total follow up period was 5 months, 1 month, half month and 2 months respectively. All of the patients were satisfied with the results. We recorded the amount of laser that was used, in addition to the total procedure time; however, these values were not found to be correlated with the size of the migrated disc.

## 4 Discussion

We report 4 cases that treated migrated lumbar disc herniation by ELND. Miraged disc material was shown directly by epiduroscope, and removed by laser for decompression of spinal nerves. Final results were good in all cases.

The epiduroscope was first introduced in the year 1931, and has developed rapidly in recent years. It is currently, a useful tool for diagnosing low back pain and is used to treat lesions in the epidural space by applying the laser as a minimally invasive intervention.^[3]^ A prospective study recently focused on predicting post-epiduroscope treatment outcomes based on epiduroscoic findings.^[4]^ This approach allows low back pain with or without leg pain to be treated more actively without surgery.

A lumbar herniated disc is a common disorder that elicits low back pain with or without radiating leg pain. Conventionally, there are 2 options for treating lumbar herniated disc: conservative treatments or surgery. Conservative treatment includes medication, nerve block (usually an epidural block), or physical therapy. The advantage of this approach is that it allows the patient to continue daily activity with less burden on both the patient, and physician, but its effectiveness is limited in some cases. Surgery has been considered the next step when conservative treatment fails to sufficiently relieve the patient's symptoms, but there are associated risks such as FBSS, and those from general anesthesia. The FBSS is defined as lumbar spinal pain of unknown origin after spine surgery, and is estimated to occur in 20 – 40% of those who receive lumbar spinal surgery.^[5]^ Multiple factors can lead to FBSS, including degenerative change of the lumbar spine after surgery, epidural adhesions, and altered biomechanics after surgery. These changes can increase tension on prevertebral and postvertebral muscles.^[6]^ FBSS is usually refractory to any treatment and is challenging for many pain physicians.^[5]^

Minimally, invasive interventional techniques have recently, been used by pain physicians to avoid complications from spine surgery for treating low back pain. ELND is a representative minimally invasive intervention approach. ELND allows physicians to visualize the epidural space directly, and to remove pathologies with direct vision, without surgery in the epidural space. The ability to immediately, remove pathologies under direct vision is very valuable; because it overcomes the limitation of conventional conservative treatments. Compared to surgery, it is advantageous because normal body structures can be conserved, and the process can be repeated if it fails. Because ELND can be performed without general anesthesia, unintended nerve injury can be prevented by observing the patient's response during the procedure, and risks from general anesthesia can be avoided.

As the device and technique have developed, the epiduroscope has become more useful for a wider variety of low back pain conditions. Especially, when using with laser, ELND can remove epidural pathologies under direct vision without surgery. Our report indicates that ELND was associated with good results for patients with chronic refractory low back, and/or lower extremity pain that could not be resolved by other conservative treatments.^[7]^ ELND also has diagnostic value because, as the name ‘epidurosope’ implies, it allows for direct visualization of conditions in the epidural space such as disc fragments; epidural vessel dilatations; adhesion; and inflammation on the dura, nerve root, and annulus. Epidural adhesions, dilated epidural vessels, and inflammation can cause back pain, which is not visible on MRI. By observing the lesion findings directly by epiduroscope, the exact cause of the pain can be determined, and the appropriate treatment plan can be developed.^[8]^ We previously, reported a case in which ELND was used to identify, and treat a real pathology, which was not found on MRI.^[9]^ Despite these advantages, there is limited evidence for the applications of ELND, and there are a limited number of reports on the subject. ELND could be indicated for various conditions with low back pain; however, additional clinical trials, and case reports are needed to obtain procedural evidence, which will help determine the indications.

One case report indicated that a combination of percutaneous endoscopic lumbar discectomy (PELD), and ELND could be applied for down-migrated disc herniation^[2]^; however, no case reports have discussed patients with migrated disc herniation that have been treated only by ELND. This report summarizes 4 cases in which migrated disc herniation was treated using only ELND. Even after the limitations of ELND were discussed, and patients were advised to undergo open surgery, the patients elected to receive ELND instead of open surgery, and they were satisfied with the results.

There is currently, no consensus on the conclusion of an ELND procedure; therefore, it is difficult, and important to decide when the procedure should be completed. Excessive laser use can lead to unintended side effects.^[10]^ When a consensus on the ending point is obtained, the limitations of the procedure should be accepted, such as the possibility of not completely, removing the pathologies from the epidural space due to very tiny instruments in a narrow space. Lasering is usually, performed until the dura is a downward move from the epiduroscopic view, and is movable, and based on the saline infusion at the upper part of the scope, which indicates spinal nerve decompression. At this point, the catheter can pass above the level of pathology, indicating sufficient space in the epidural area.

## 5 Conclusion

In the cases in this report, we were able to mechanically, remove the disc material that compressed the spinal nerve through the ENLD procedure, and the patients’ symptoms were relieved with satisfaction. ELND provided sufficient treatment for lumbar migrated herniated disc for patients that did not want to undergo open spine surgery. However, the slow learning curve, and the high costs are currently barriers to the procedure. We hope that more cases of ELND will be reported, and we would like to determine appropriate indications of ELND based on the case reports.

## Acknowledgments

The authors have no funding to disclose for this paper and thank all the anonymous reviews and editors for their suggestions, which will be helpful for the authors to improve the paper.

## Author contributions

**Conceptualization:** D. Jo.

**Data curation:** J. Oh.

**Writing – original draft:** J. Oh.

**Writing – review & editing:** D. Jo.
